# An experimental and theoretical characterization of the electronic structure of doubly ionised disulfur

**DOI:** 10.1038/s41598-022-16327-8

**Published:** 2022-07-18

**Authors:** Emelie Olsson, Tarek Ayari, Veronica Ideböhn, Måns Wallner, Richard J. Squibb, Jonas Andersson, Andreas Hult Roos, Stefano Stranges, John M. Dyke, John H. D. Eland, Majdi Hochlaf, Raimund Feifel

**Affiliations:** 1 Department of Physics, University of Gothenburg, Origovägen 6B, 412 58 Gothenburg, Sweden; 2 Université Gustave Eiffel, COSYS/IMSE, 5 Bd Descartes, 77454 Champs sur Marne, France; 3 ELI Beamlines, Institute of Physics AS CR, v.v.i., Na Slovance 2, 182 21 Prague 8, Czech Republic; 4 IOM-CNR Tasc, SS-14, Km 163.5 Area Science Park Basovizza, 34149 Trieste, Italy; 5 Dipartimento di Chimica e Tecnologie del Farmaco, Universitá Sapienza, 00185 Rome, Italy; 6 School of Chemistry, University of Southampton, Highfield, Southampton, SO17 1BJ UK; 7 Department of Chemistry, Physical and Theoretical Chemistry Laboratory, Oxford University, South Parks Road, Oxford, OX1 3QZ UK

**Keywords:** Atomic and molecular interactions with photons, Electronic structure of atoms and molecules, Chemical physics

## Abstract

Using time-of-flight multiple electron and ion coincidence techniques in combination with a helium gas discharge lamp and synchrotron radiation, the double ionisation spectrum of disulfur (S$_2$) and the subsequent fragmentation dynamics of its dication are investigated. The S$_2$ sample was produced by heating mercury sulfide (HgS), whose vapour at a suitably chosen temperature consists primarily of two constituents: S$_2$ and atomic Hg. A multi-particle-coincidence technique is thus particularly useful for retrieving spectra of S$_2$ from ionisation of the mixed vapour. The results obtained are compared with detailed calculations of the electronic structure and potential energy curves of S$_2^{2+}$ which are also presented. These computations are carried out using configuration interaction methodology. The experimental results are interpreted with and strongly supported by the computational results.

## Introduction

Disulfur, S$_2$, is a reactive intermediate molecular species, which is of great interest from both a fundamental perspective, due to its similarity to molecular oxygen, and because it is important in a number of other scientific areas. In particular, it is astrophysically significant, and is especially important in the atmospheres of Jovian planets. Disulfur is a known constituent of the atmosphere of Io, a moon of Jupiter, in the plume above Mt. Pele^1^. Other charged sulfur ions are also known to exist in the torus around Europa^2^ and in the ionosphere of Jupiter^3^. Furthermore, S$_2$ is a component of Comet IRAS–Araki–Alcock (1983 d)^4,5^ and of the coma of comet 67P/Churyumov–Gerasimenko^6^, but its origin in comets, for which several scenarios were proposed^7^, is not yet known. Disulfur also plays a significant role in magmatic and volcanic processes on Earth^8^. Apart from that, disulfide bonds are very important in the stabilisation of protein structures^9^.

The S$_2$ molecule has 12 valence electrons, derived from the $3s^{2}3p^{4}$ valence electrons of each S atom. S$_2$ has the following ground state electronic configuration, where some of the core shell orbitals are omitted for simplicity:

$$\ldots1\sigma ^{2}_{\mathrm {g}} 1\sigma ^{2}_{\mathrm {u}} 2\sigma ^{2}_{\mathrm {g}} 2\sigma ^{2}_{\mathrm {u}} 3\sigma ^{2}_{\mathrm {g}} 1\pi ^{4}_{\mathrm {u}} 1\pi ^{4}_{\mathrm {g}} 3\sigma ^{2}_{\mathrm {u}} 4\sigma ^{2}_{\mathrm {g}} 4\sigma ^{2}_{\mathrm {u}} 5\sigma ^{2}_{\mathrm {g}} 2\pi ^{4}_{\mathrm {u}} 2\pi ^{2}_{\mathrm {g}}$$

As in molecular oxygen, the neutral ground state has $\,^3\Sigma ^-_{\mathrm {g}}$ symmetry, the first excited state is $\,^1\Delta _{\mathrm {g}}$ and the second excited state is $\,^1\Sigma ^+_{\mathrm {g}}$. The ground state of the doubly charged ion comes from removal of the two outermost $\pi _{\mathrm {g}}$ electrons and so is a $\,^1\Sigma ^+_{\mathrm {g}}$ state. The single ionisation electron spectrum of S$_2$ reported by Dyke et al.^10,11^ was compared with spectra at 21.22 eV photon energy measured in the present work and used with other evidence to verify the presence of S$_2$. Dyke et al.^10^ found the lowest single ionisation energy to be 9.38 eV, confirmed by more recent studies by Hrodmarsson et al.^12^. According to the rule-of-thumb for double ionisation^13^, $\mathrm {DIE} = (2.2\pm {0.03})\mathrm {IE}+(11.5\pm {1})/r_{12}$, and using an internuclear distance of 1.889 Å^14^, we expect the lowest double ionisation energy of S$_2$ to be approximately 26.5 eV. However, electron-impact experiments of sulfur performed by Zavilopulo et al.^15^ previously reported a value of 16.8 eV for the double ionisation energy of S$_2$. If we assume this is instead the second ionisation energy, the sum of the first and second ionisation energy suggests a double ionisation energy of 26.2 eV.

In this paper, we present measurements of single-photon double ionisation electron spectra of S$_2$, thus characterising the electronic structure of S$_2^{2+}$ for the first time. The experimental spectra are obtained by irradiating the vapour produced from heated mercury sulphide (HgS), whose main constituents at suitable temperatures are S$_2$ and atomic Hg^16^. For the experiments, we used both monochromatised He II emission (primarily 40.81 eV) provided by a home-laboratory gas-discharge lamp and wave-length selected soft X-rays (90 and 180 eV) provided by a synchrotron radiation facility. Whereas the 40.81 eV gives higher spectral resolution for the ground and the first excited dicationic states, the 90 eV photon energy is chosen to obtain a more complete valence double ionisation spectrum. Moreover, using 180 eV implies double ionisation primarily by intermediate core hole formation followed by Auger decay, and can thus be expected to result in a dication spectrum of different intensity distribution. Because atomic Hg gives very strong single and double ionisation signals and is highly abundant in these experiments, multi-particle coincidence experimental and analysis techniques are essential to select the double ionisation electron spectra of S$_2$.

To assist interpretation of the results, we computed the potential energy curves of the lowest states of S$_2^{2+}$ using a multi-configuration approach. We thus deduced the vertical double ionisation energies of S$_2$ and determined a set of spectroscopic constants for the (meta)stable states of S$_2^{2+}$. Additionally, the potential energy curves of S$_2$ and S$_2^{2+}$ were calculated, for validation and to deduce the first adiabatic double ionisation energy of S$_2^{2+}$. Comparison of the detailed calculations of the electronic structure, potential energy curves and energetics of S$_2^{2+}$ with the experimental results confirms the formation of this dication upon doubly photoionising the vapour over heated HgS.

## Results and discussion

**Figure 1:**
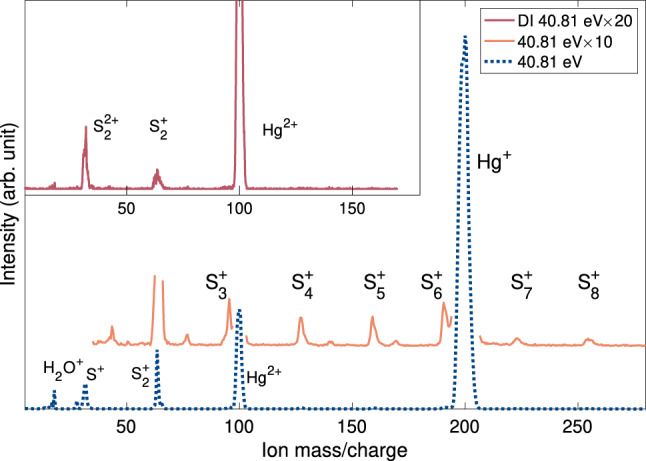
Full mass spectrum of the vapour of heated HgS at 40.81 eV (dashed blue line), with $\times$10 magnification (solid orange line), and the spectrum of ions extracted in coincidence with two electrons (solid red line). The spectra are dominated by Hg, followed by S$_2$.

Figure 1 shows three variants of the mass spectrum of mercury sulfide vapour obtained at the photon energy of 40.81 eV. The lower spectrum (dashed blue line) includes all ions produced at this energy and is dominated by single and double ionisation signals of Hg as well as S$_2^+$ and S$^+$/S$_2^{2+}$. The middle (solid orange line) spectrum shows the same spectrum magnified by a factor of 10, and with the intensities of the strongest peaks truncated, so as to emphasise the weaker features. Here, we can also identify several sulfur oligomers, up to S$_8^{+}$. Inspection of the single ionisation electron spectrum shows that S$_2$ is produced mainly in its $^3\Sigma ^-_{\mathrm {g}}$ ground state^10^, but a small proportion of excited molecules in the $\,^1\Delta _{\mathrm {g}}$ state, about 0.5 eV higher in energy^17^ cannot be ruled out. The upper (solid red line) spectrum in Fig. 1 shows ions extracted in coincidence with two electrons, where the singly charged species must originate from charge-separation processes of nascent doubly charged molecular species.

**Figure 2:**
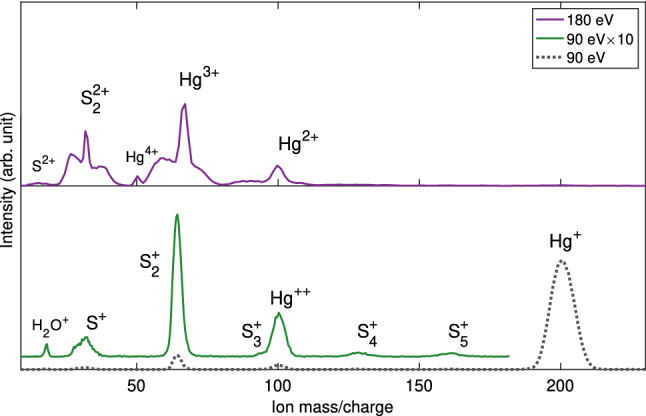
Lower panel: Mass spectra reflecting primarily singly charged ions, which were extracted in coincidence with one electron of a binding energy of 5–20 eV, obtained at the photon energy of 90 eV (dashed grey line and solid green line). The spectrum in dashed grey is dominated by Hg$^+$. The green plot is $\times$10 magnification of that part of the spectrum which is relevant for the discussion. The full-width-half-maximum of the S$^+$ signal suggests substantial contributions from fragmentation of S$_2^{2+}$. Upper panel: a mass spectrum reflecting ions detected in coincidence with two electrons, obtained at the photon energy of 180 eV (solid purple line). At this photon energy, the double ionisation route by Auger decay of the S 2p core holes is dominant.

Figure 2 shows similar spectra obtained using synchrotron radiation. The lower (dashed grey line) mass spectrum shows all single ions in coincidence with single electrons with binding energy in the range 5–20 eV, obtained at a photon energy of 90 eV, which is again dominated by ionisation of Hg. The 10$\times$ enlargement (solid green line) reflects ionisation of several sulfur species. We note that the S$^+$ feature also contains S$_2^{2+}$, and from its width and shape seems to be dominated by fragmentation of S$_2^{2+}$. The upper (solid purple line) spectrum was obtained at 180 eV and contains both Hg and sulfur species, in particular a strong signal of S$^+$ / S$_2^{2+}$. In this case, the ions were filtered by selection of the majority of double ionisation events from Auger decay of an initial S 2p hole, selected on the photoelectron kinetic energy range of 6–12 eV. At this photon energy a minority of electrons also comes from valence double ionisation^18,19^, and from multiple Auger decays producing triple and quadruple ionisation, explaining the presence of multiply charged ions in the spectrum. At slightly higher photon energy (not shown) core-valence ionisation^20^ can also contribute.

Since the species S$_2^{2+}$ and S$^{+}$ have the same mass to charge ratio ($\mathrm {m/q}$ = 32), their features coincide in the mass spectra. S$^{+}$ originating from dissociation of the dimer dication, S$_2^{2+}$ $\xrightarrow {}$ S$^{+}$ $+$ S$^{+}$, will have a kinetic energy release, which will lead to a broadening of the spectral feature. This broadening is very substantial in the 180 eV mass spectra, leading to the formation of distinguishable, separated ‘wings’. However, the sharper centre of the peak at $\mathrm {m/q}$ = 32, which corresponds to ions with little to no initial kinetic energy, could also be contaminated by contributions from nascent atomic S$^{+}$ produced directly by the source. Hence, the peak at $\mathrm {m/q}$ = 32 in the unfiltered spectrum (dashed grey line) may contain contributions from atomic S$^{+}$, from fragmentation of S$_2^{2+}$, and possibly S$^{+}$ from fragmentation of more highly charged S$_2$ ions or higher mass sulfur oligomers.

To determine how much atomic S might be present in the vapour, the relative intensity of the peak at $\mathrm {m/q}$ = 32 can be compared to Hg$^{2+}$ in Fig. 1. The intensity difference for the Hg$^{2+}$ peak between the dashed blue and the solid red spectra could, in principle, be affected by possible differences in accidental coincidences, but is more likely to be affected by the collection-detection efficiencies when selecting one extra electron. Any contribution from atomic S at $\mathrm {m/q}$ = 32 is removed by selecting events with two electrons (solid red line) which makes it possible to estimate the amount of atomic S in the full spectrum by comparison to Hg$^{2+}$. S$^{+}$ originating from dissociation will have kinetic energy release, thus broadening the corresponding spectral feature and giving rise to the intensity on either side of the $\mathrm {m/q}$ = 32 peak. The conclusion is that at least half the intensity in the $\mathrm {m/q}$ = 32 peak in the full spectrum must be associated with dissociation of S$_2^{2+}$.

According to previous studies of HgS vapour, the fraction of atomic S increases with sample temperature^16^, and we would therefore expect to see a greater fraction of atomic S in the vapour for the higher temperature (320 $^\circ$C) data sets compared to the lower temperature (280 $^\circ$C) data sets. The higher temperature data sets were obtained at 90 eV and 180 eV, whose mass spectra are shown in Fig. 2. Here, the dashed grey and solid green spectra comprise events involving one ion and one electron, and therefore includes contributions from all single ionisation processes. Double ionisation can also be seen in these spectra if only one of the emitted electrons is detected, and this is why we see doubly charged Hg. The peak at $\mathrm {m/q}$ = 32 in these spectra could then be expected to include contributions from ionised atomic S, S$_{2}^{2+}$ or S$^{+}$ from oligomer dissociation. However, the broad width of the peak and lack of a sharp, central feature suggests there is little contribution from low kinetic energy ions, and instead comes predominantly from molecular dissociation, implying that there is hardly any atomic S present in the mass spectra.

Conversely, for spectra where only ions detected in coincidence with two electrons were extracted (e.g. the purple spectrum taken at 180 eV), there should be hardly any contribution from nascent S since the emission of two electrons from this species would result in a S$^{2+}$ final state ($\mathrm {m/q}$ = 16). In these cases, ions of $\mathrm {m/q}$ = 32 must come almost exclusively from charge-retaining dissociation events of molecular sulfur species. The exception would be in the case where there are significant differences in the accidental coincidences; however, we expect this to be unlikely given our aforementioned coincidence conditions. We can therefore ensure the removal of any contributions from nascent S by restricting our data set to two-electron processes that are coincident with an ion at $\mathrm {m/q}$ = 32.

**Figure 3:**
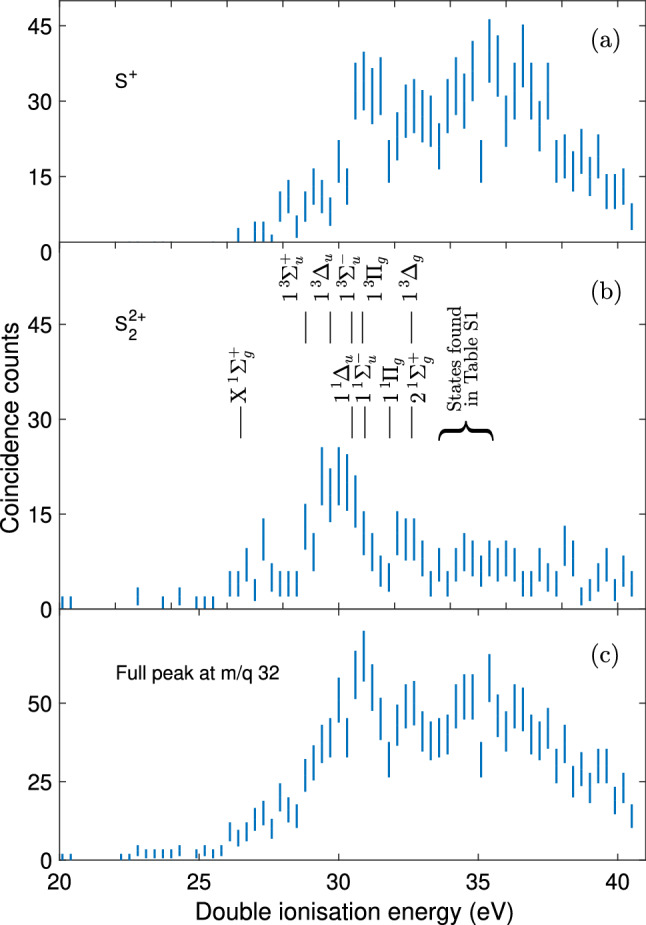
Double ionisation electron spectra from electron-ion coincidences at the photon energy of 40.81 eV. Each spectrum is based on electron pairs extracted in coincidence with all ions at mass to charge $\mathrm {m/q}$ = 32 (**c**), the S$_2^{2+}$ ion (**b**) or one of its S$^{+}$ fragments (**a**). Error bars represent the statistical uncertainty of the included coincidence counts. The middle panel shows calculated vertical excitation energies from the ground state of neutral S$_2$. All calculated states can be found in Supplementary Table S1.

The double ionisation spectra of the S$_2^{2+}$ and the S$^{+}+$ S$^{+}$ species can be obtained by selecting the two electrons in coincidence with the corresponding ion, and then subtracting the kinetic energy sum of the two electrons from the known photon energy. These spectra are shown in Fig. 3a for S$^{+}$ and (b) for S$_2^{2+}$ for a photon energy of 40.81 eV. The bottom panel (c) shows the complete double ionisation spectrum, where the two electrons were correlated with the entire ion peak centred around $\mathrm {m/q}$ = 32. For the (b) and (a) panels, electron pairs associated primarily with S$_2^{2+}$ or S$^{+}$ have been extracted, by selecting the narrow part of the ion peak and the higher kinetic energy wings, respectively. We also expect some contribution from S$^{+}$ in the narrow part of the peak, but subtraction of spectrum Fig. 3a from b shows no appreciable difference.

From the S$_{2}^{2+}$ spectrum seen in Fig. 3b, it is apparent that the lowest double ionisation energy is located around 27 eV where a first, comparatively sharp feature is observed, while the most intense peak is observed very near 30 eV. The maximum of the first peak corresponds to the vertical double ionisation energy of S$_2$, and the non-zero signal starting slightly above 26 eV may correspond to the adiabatic double ionisation energy of S$_2$. These values are likely to differ since non-favorable Franck–Condon factors upon doubly ionising S$_2$ are expected (see explanation below). Based on the FWHM of the Hg$^{2+}$ $^1$S$_0$ state in the double ionisation spectrum, the estimated resolution at 40.81 eV is 0.8 eV.

For the S$^{+}$ spectrum shown in Fig. 3a, the appearance energy is somewhat higher, nearer to 30 eV, with the first strong feature at about 31 eV. With the single ionisation energy of atomic S from NIST^21^ and the dissociation energy of S$_2$ from Sun et al.^17^, the thermodynamic threshold for S$^{+}$ $+$ S$^{+}$ from S$_2$ is 25.14 eV. This implies an excess energy of 5 eV at 30 eV appearance energy, which could be mainly kinetic energy, possibly with some excitation of the atomic sulfur ions. A kinetic energy release of this magnitude is confirmed by calculations based on the width of the $\mathrm {m/q}$ = 32 ion peak in the time of flight spectra and simulations of the electric field in the interaction region.

**Figure 4:**
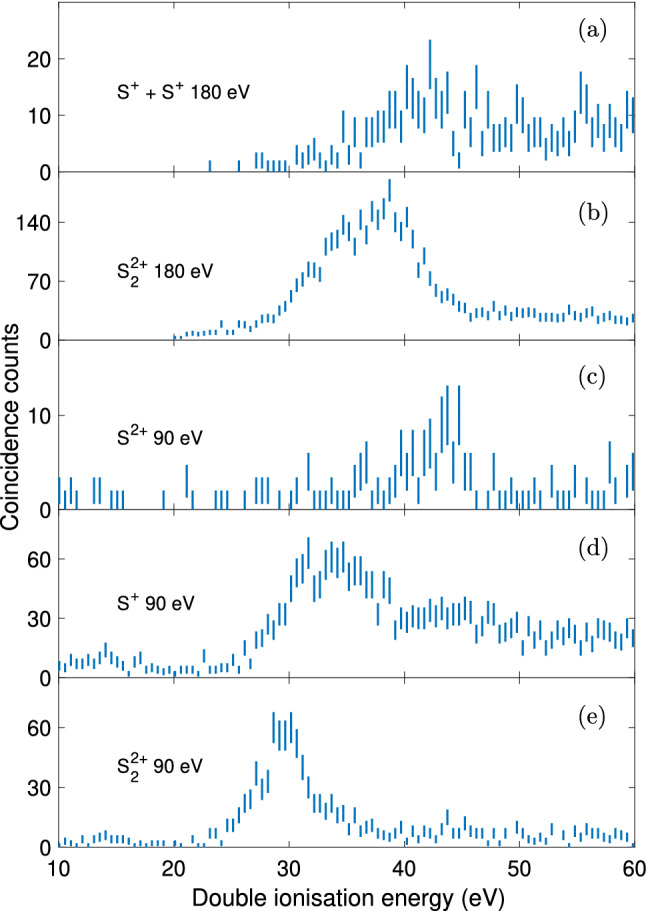
Double ionisation electron spectra obtained at the photon energies of 90 and 180 eV, based on electron pairs in coincidence with S$_2^{2+}$, (**b**) and (**e**), S$^{+}$, (**a**) and (**d**), or S$^{2+}$ signals, (**c**). Error bars show only the statistical uncertainty of the included coincidence counts.

For comparison, Fig. 4 shows the double ionisation spectra of the vapour of mercury sulfide obtained at the photon energies of 90 eV and 180 eV, respectively. The spectra show electron pairs selected in coincidence with ions, either on the narrow central peak of the $\mathrm {m/q}$ = 32 feature in Fig. 2, primarily representing S$_2^{2+}$ species, or the wings around $\mathrm {m/q}$ = 32 primarily associated with S$^{+}$ species. The relative intensities of the different spectra are to be taken with caution, since some of the high kinetic energy S$^{+}$ ions may had high enough off-axis velocity components to hit sufaces and so evade detection. Also, the shapes of the features at the two photon energies are not expected to be the same, partly because energy resolution is better at the lower photon energy, but more importantly because the ionisation processes are distinct in each case. At 90 eV, ionisation can be expected to be entirely from the valence shell, whereas at 180 eV double ionisation via Auger decay of a S 2p hole is dominant. The estimated resolution, based on the full width half maximum (FWHM) of the Hg$^{2+}$ $^1$S$_0$ state in the double ionisation spectrum, is 3 eV at 90 eV and 6 eV at 180 eV. From the S$_{2}^{2+}$ spectrum at 90 eV, a weak feature can again be discerned at 27.2 eV, whereas a stronger peak is observed around 30 eV. Electron pairs extracted in coincidence with S$^+$ again show a shift of the onset towards higher ionisation energies compared to the S$_2^{2+}$ spectrum, akin to the 40.81 eV measurements described above. Also, in the 90 eV electron pair spectrum selected on $\mathrm {m/q}$ = 16 which corresponds to the S$^{2+}$ species, the signal starts around 40 eV. The formation of S$^{2+}$ can be expected to originate from charge-retaining fragmentation of S$_2^{2+}$. Such charge-retaining fragmentation could occur from more highly excited states, which is in line with its onset around 40 eV. If we assume that the S$^{2+}$ + S fragments are formed in their electronic ground states, this locates this asymptote at 38.13 eV i.e. somewhat below this onset. We obtain a better agreement if we consider that the S atom is formed in the $^1$D state instead.

For the data obtained at the photon energy of 180 eV, the double ionisation electron spectra extracted in coincidence with S$_2^{2+}$ and S$^{+}$ can be seen in Fig. 4a,b, respectively. For Fig. 4a, the spectrum is based on fourfold coincidences, with two S$^{+}$ ions and two electrons, in contrast to all other coincidence spectra shown in this figure which are based on one ion and two electrons. The degradation in resolution at these comparatively high electron kinetic energies does not allow any sharp peaks to be identified, but the overall appearance of the spectra matches that of the 90 eV data. For the S$^{+}$ + S$^{+}$ channel, the high double ionisation energy explains the very high kinetic energy release we see in the mass spectrum (cf. Fig. 2, upper panel).

**Figure 5:**
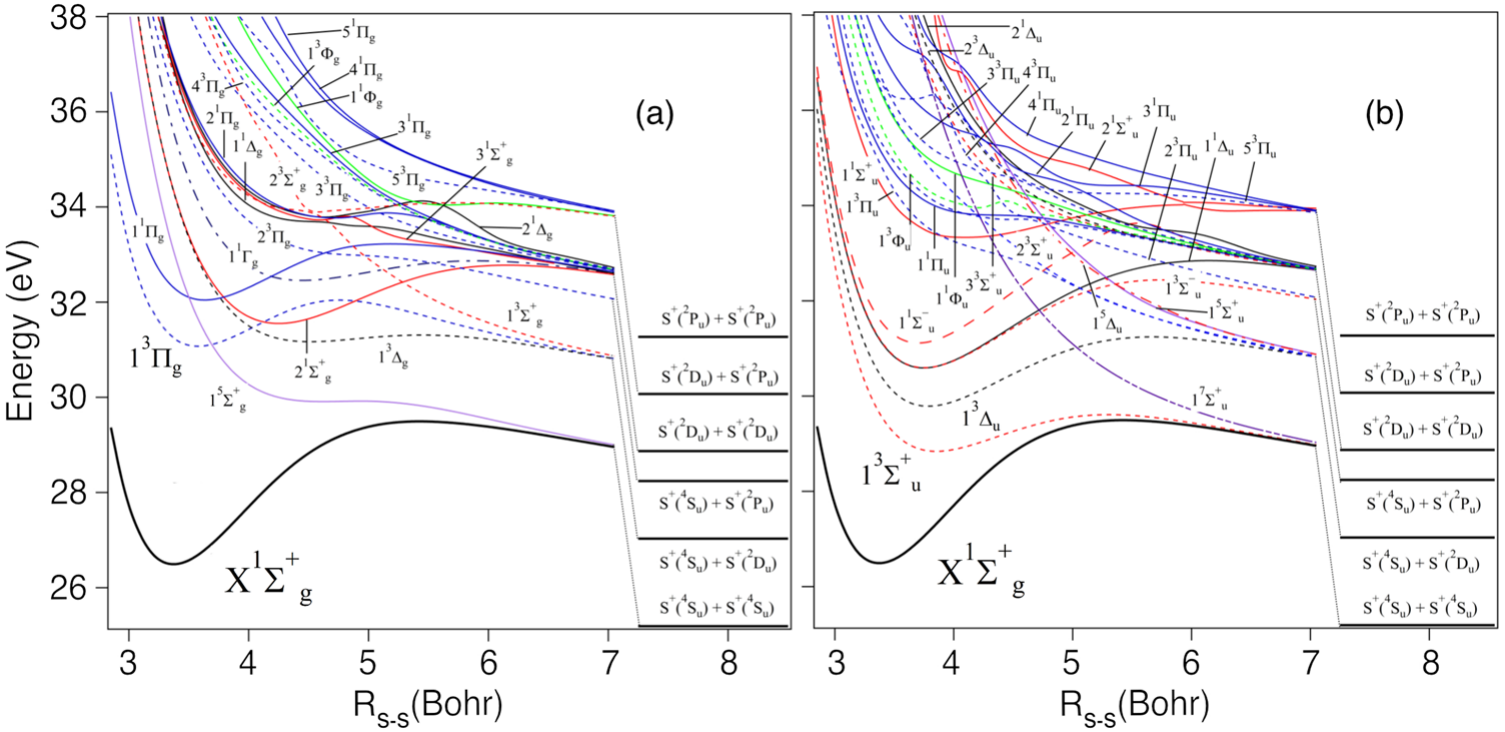
Computed MRCI/aug-cc-pV(5+d)Z potential energy curves for S$_2^{2+}$ of (**a**) gerade symmetry and (**b**) ungerade symmetry. The reference energy is that of S$_2^{2+}$(X$^{1}\Sigma ^{+}_{\mathrm {g}}$) at the equilibrium bond distance.

For the interpretation of the experimental results, we make use of our ab initio computations on the potential energy curve of the neutral S$_2$ (X$^{3}\Sigma _{\mathrm {g}}^-$) ground state and the potential energy curves of the lowest electronic states of the S$_2^{2+}$ dication. The theoretical results are presented in Table 1, Supplementary Table S1 and as potential energy curves for the gerade and ungerade states in Fig. 5a,b, respectively. For the dication, the potential energy curves result in bound electronic states for lower double ionisation energies, whereas for higher double ionisation energies the states are unbound which lead to efficient dissociation. In particular, a relatively deep potential well is computed for the ground electronic state of S$_2^{2+}$ confirming the long lived nature of this dicationic state and observation of a peak in the mass spectra associated with S$_2^{2+}$. The formation of S$_2^{2+}$ (X$^{1}\Sigma _{\mathrm {g}}^+$) is associated with the removal of two electrons from the outermost $2\pi ^{2}_{\mathrm {g}}$ molecular orbital, which is antibonding in nature. This results in a shortening of the S–S equilibrium distance. For instance, we calculated a S–S distance of 3.588 Bohr (= 1.898 Å) for S$_2$ (X$^{3}\Sigma _{\mathrm {g}}^-$) and of 3.372 Bohr (= 1.784 Å) for S$_2^{2+}$ (X$^{1}\Sigma _{\mathrm {g}}^+$) (cf. Table 2). Therefore, non-favorable Franck–Condon factors are expected for the S$_2$ (X$^{3}\Sigma _{\mathrm {g}}^-$) $\xrightarrow {}$ S$_2^{2+}$ (X$^{1}\Sigma _{\mathrm {g}}^+$) + 2 e$^-$ transition. In particular, the adiabatic double ionisation energy of S$_2$ should be difficult to measure from the experimental spectra due to the lack of vibrationally resolved structures in contrast to the threshold photoelectron coincidence spectrum and the complete valence double ionisation electron spectrum of O$_2$^22,23^.

**Table 1:** Computed dissociation limits for S$_2^{2+}$ leading to two S$^{+}$ fragments in their ground and excited states $^{4}$S$_{\mathrm {u}}$, $^{2}$D$_{\mathrm {u}}$ and $^{2}$P$_{\mathrm {u}}$ arising from the $3s^{2}3p^{3}$ configuration. The lowest asymptote is located experimentally as 25.14 eV above the vibrational ground state of neutral S$_2$^17,21^. The adiabatic double ionisation energy of S$_2$ is calculated as 26.31 eV, lying 1.18 eV above the lowest asymptote. The reference energy is that of S$_2$ (X$^{3}\Sigma _{\mathrm {g}}^{-}$) $v = 0$.

Dissociation fragments	Energy (eV)	Molecular states
S$^+$($^{4}$S$_{\mathrm {u}}$) + S$^+$($^{4}$S$_{\mathrm {u}}$)	25.14	$^{1}\Sigma _{\mathrm {g}}^{+}$, $^{3}\Sigma _{\mathrm {u}}^{+}$, $^{5}\Sigma _{\mathrm {g}}^{+}$, $^{7}\Sigma _{\mathrm {u}}^{+}$
S$^+$($^{4}$S$_{\mathrm {u}}$) + S$^+$($^{2}$D$_{\mathrm {u}}$)	26.98	$^{3,5}(\Sigma ^{+},\Pi ,\Delta )_{u,g}$
S$^+$($^{4}$S$_{\mathrm {u}}$) + S$^+$($^{2}$P$_{\mathrm {u}}$)	28.19	$^{3,5}(\Sigma ^{-},\Pi )_{u,g}$
S$^+$($^{2}$D$_{\mathrm {u}}$) + S$^+$($^{2}$D$_{\mathrm {u}}$)	28.82	$^{1}(\Sigma ^{+}_{\mathrm {g}}(3), \Sigma ^{-}_{\mathrm {u}}(2), \Pi _{\mathrm {g}}(2),\Pi _{\mathrm {u}}(2), \Delta _{\mathrm {g}}(2), \Delta _{\mathrm {u}}, \Phi _{\mathrm {g}}, \Phi _{\mathrm {u}}, \Gamma _{\mathrm {g}})$,
		$$^{3}(\Sigma ^{+}_{\mathrm {u}}(3), \Sigma ^{-}_{\mathrm {g}}(2), \Pi _{\mathrm {g}}(2),\Pi _{\mathrm {g}}(2), \Delta _{\mathrm {g}}, \Delta _{\mathrm {u}}(2), \Phi _{\mathrm {g}}, \Phi _{\mathrm {u}}, \Gamma _{\mathrm {u}})$$
S$^+$($^{2}$D$_{\mathrm {u}}$) + S$^+$($^{2}$P$_{\mathrm {u}}$)	30.02	$^{1,3}(\Sigma ^{+}, \Sigma ^{-}(2), \Pi (3),\Delta (2), \Phi )_\mathrm {u,g}$
S$^+$($^{2}$P$_{\mathrm {u}}$) + S$^+$($^{2}$P$_{\mathrm {u}}$)	31.22	$^{1}(\Sigma ^{+}_{\mathrm {g}}(2), \Sigma ^{-}_{\mathrm {u}}, \Pi _{\mathrm {g}},\Pi _{\mathrm {u}}, \Delta _{\mathrm {g}})$,
		$$^{3}(\Sigma ^{+}_{\mathrm {u}}(2), \Sigma ^{-}_{\mathrm {g}}, \Pi _{\mathrm {g}},\Pi _{\mathrm {u}}, \Delta _{\mathrm {u}})$$

The empirical rule-of-thumb for estimating double ionisation energies presented by Molloy et al.^13^, $\mathrm {DIE} = (2.2\pm {0.03})\mathrm {IE}+(11.5\pm {1})/r_{12}$, and using the values of 9.38 eV for the lowest single ionisation energy of S$_2$^10^ and the equilibrium bond distance of 1.889 Å for neutral S$_2$^14^, gives $26.7\,\pm \,{0.8}$ eV; this agrees with our experimental double ionisation energy of about 27 eV. Besides, our calculations predict the first adiabatic double ionisation energy for S$_2$ to be 26.31 eV and the vertical double ionisation energy to be at 26.49 eV. Experimentally, we observe a weak feature at $27.2\,\pm \,{3}$ eV (cf. Fig. 4e) for the S$_2^{2+}$ species at 90 eV, while the first signal of the S$_2$ double ionisation at 41 eV can be seen at $27.3\,\pm \,{0.8}$ eV (cf. Fig. 3b). This feature is not discernible at the photon energy of 180 eV, most likely because of the lower resolution for electrons with higher kinetic energies. The present calculations allow assignment of this feature in the double ionisation spectrum of S$_2$ to the S$_2^{2+}$ ground state.

According to the potential energy curves (cf. Fig. 5a,b), the ${}^{1}\Sigma ^+_{\mathrm {g}}$ ground state for the S$_2^{2+}$ correlates directly to ground state products. It is separated towards the S$^+$ + S$^+$ fragments by a potential barrier of about 3 eV. This leads to a computed appearance energy of 29.3 eV and a kinetic energy release of 4.3 eV. Both values are in line with the experimental data. Figure 3a suggests that the S$_2^{2+}$ ion is stable to about 30 eV, between the ${1}^{3}\Delta _{\mathrm {u}}$ and the ${1}^{3}\Pi _{\mathrm {g}}$ dicationic states, and a kinetic energy release of about 5 eV is deduced from the width of the $\mathrm {m/q}$ = 32 in Fig. 1.

The equilibrium bond distance in neutral S$_2$ is 3.588 Bohr (= 1.898 Å), so according to the calculated potential energy curves, vertical double ionisation can access the first excited state at about 29 eV (${1}^{3}\Sigma ^+_{\mathrm {u}}$), which could explain the shoulder of the larger peak in Fig. 3b, at $28.8 \pm 0.8$ eV. The next excited state is ${1}^{3}\Delta _{\mathrm {u}}$ at about 30 eV, which might contribute to the feature we see for S$_2^{2+}$ in both Fig. 4e at $29 \pm 3$ eV and Fig. 3c at $30.0 \pm 0.8$ eV. This peak is also present in Fig. 3b, but less easily discernible. The ${1}^{3}\Delta _{\mathrm {u}}$ dicationic state has an equilibrium bond length close to the equilibrium bond length of neutral S$_2$, and could produce a narrow peak. According to the potential energy curves, this state does not correlate to ground state products, but the S$_2^{2+}$ (1$^{3}\Delta _{\mathrm {u}}$) state can be predissociated by the (1$^7\Sigma _{\mathrm {u}}^+$) state involving spin-orbit interaction at their crossing (i.e. for energies of $\sim$ 4.5 eV above S$_2^{2+}$ (X$^1\Sigma _{\mathrm {g}}^+$)). The septet state correlates adiabatically to the lowest dissociation limit. We deduce from the calculations an appearance energy of $\sim$ 30.8 eV and a kinetic energy release of $\sim$ 4.8 eV. This could be the dissociation which we see in Fig. 3a around 31 eV.

Apart from that, our calculations also predict a ${1}^{3}\Pi _{\mathrm {g}}$ dicationic state near 31 eV with an equilibrium bond length similar to that of neutral S$_2$. This state is another candidate giving rise to the feature around 31 eV in the S$^{+}$+ S$^{+}$ channel, in both the 40.81 eV (see Fig. 3a) and 90 eV (see Fig. 4d) spectra. In comparison to O$_2$ double photoionisation, whose most prominent sharp feature is an analogous $^3\Pi _{\mathrm {g}}$ state, we consider the ${1}^{3}\Pi _{\mathrm {g}}$ more likely to be responsible for the main peak seen at $30.9 \pm 0.8$ eV in the spectra (see Fig. 3a,c)^24,25^. Another similarity between the S$_2$ and O$_2$ double ionisation spectra is the distinct separation between the ground state ${}^{1}\Sigma ^+_{\mathrm {g}}$ and the first excited states^24^.

## Conclusions

We have presented a combined experimental and theoretical study on the double ionisation of disulfur. In particular, we measured the first double ionisation electron spectrum of disulfur, S$_2$, which allowed us to determine the lowest double ionisation energy of this reactive intermediate. The double ionisation electron spectrum of S$_2$ showed many similarities with that of O$_2$, but with different energy separations between the states. The experimental results obtained from our electron-ion coincidence measurements at 40.81 eV and 90 eV showed very similar spectral structures for the two photon energies, revealing a vertical double ionisation energy of 27.3 ± 0.8 eV. This lowest double ionisation energy is strongly supported by the empirical rule-of-thumb for the lowest double ionisation energy and by ab initio calculations. Also, in the double ionisation spectrum a weakly excited first excited state at 28.8 ± 0.8 eV was identified as ${1}^{3}\Sigma ^+_{\mathrm {u}}$ and a stronger peak near at 30.0 ± 0.8 eV was identified as the dicationic ${1}^{3}\Delta _{\mathrm {u}}$ state. For the charge separated decay, the most prominent feature at 30.9 ± 0.8 eV was identified as a ${1}^{3}\Pi _{\mathrm {g}}$ state. The findings of the present work are expected to contribute to the understanding of the possible role of the presence of dications on the escape of molecules from interstellar atmospheres, a key topic related to the evolution of planets. We note that especially fragment ions generated by charge separating dissociation of molecular dications may possess sufficiently high kinetic energy to allow the fragments to achieve escape velocity, thus having an impact on the erosion of the atmosphere. In addition, these energetic fragments can initiate reactions, which are endothermic for cold fragment ions, processes which may also be significant in the interstellar medium and planetary atmospheres.

## Methods

### Experimental details

The experiments were performed in our laboratory at the University of Gothenburg and at the UE52-SGM beamline at the BESSY-II storage ring of the Helmholtz-Zentrum in Berlin, where our magnetic bottle electron spectrometer was augmented with an in-line ion time-of-flight spectrometer, similarly configured as presented previously in Refs.^26,27^. Utilising multi-coincidences of electrons and ions, it is possible to select specific ions when extracting electron pairs originating from double ionisation of S$_2$, thus rigorously eliminating contamination from Hg.

In the experimental set-up used, a ring magnet with a hollow conical pole-piece, located about 10 mm away from the light matter interaction point, provides a divergent magnetic field which collects essentially all electrons emitted in a solid angle of 4$\pi$. A solenoid surrounding an about 2.2 m long electron flight tube creates a homogeneous field which guides the electrons towards a micro-channel plate detector at the end of the tube. The detector registers the flight time of the electrons relative to the pulsed light source, which are converted to energies based on an established calibration procedure relying on known single ionisation and autoionisation energies described below. The electron kinetic energy resolution for this setup is typically $\Delta E$ $\approx$ *E*/20 and the overall collection-detection efficiency is about 50%^26^. A few hundred nanoseconds after photoionisation, once the electrons have escaped the interaction region, an electric field is applied across the interaction region in order to accelerate the ions through the ring magnet in the opposite direction to the electrons. The ions travel through a 0.12 m long flight tube towards a second micro-channel plate detector. The electric fields are optimised to obtain Wiley–McLaren time focusing conditions^28^. The flight time for the ions is proportional to $\sqrt{m/q}$, where *m*/*q* is the mass to charge ratio. The mass resolution is about $\Delta m$ $\approx$ *m*/40 for thermal ions and the ion collection-detection efficiency is about 10%. The number of accidental coincidences, where ions and electrons from different ionisation events are detected together, is reduced by keeping the ionisation rate in the interaction region below 2%.

At the University of Gothenburg, a helium gas discharge lamp was used as light source, providing few-nanosecond pulses of both He I and He II radiation of sufficiently high flux at a repetition rate of about 4 kHz. The radiation was monochromatized with a grating, and the He II$\alpha$ atomic emission line at 40.81 eV was used for the double ionisation measurements. The synchrotron radiation experiments were performed at the BESSY-II storage ring operating in single-bunch mode, at the photon energies of 90 eV and 180 eV. Single bunch operation is critical for this type of experiment, because it has a time-separation of the radiation pulses of 800.5 ns (1.25 MHz), which, in turn, allows us to employ a mechanical chopper system to further reduce the pulse repetition rate^29^ to about 10 kHz as required by our multi-electron-ion coincidence technique.

In the light-matter interaction region of our spectrometer the radiation pulses intercept the effusive vapour produced by heating mercury(II) sulfide red (Sigma Aldrich 243566-50G) in a resistively heated oven. The oven was operated at a temperature of 320 $^{\circ }$C at the synchrotron radiation facility and at 280 $^{\circ }$C in Gothenburg. The lower temperature proved preferable, since the vapour had similar composition to that produced at higher temperatures, but resulted in greater sample longevity.

In Gothenburg, the single ionisation electron spectra of molecular oxygen obtained at 21.22 and 40.81 eV were used for the kinetic energy calibration of the time-of-flight electron spectrometer, together with autoionisation energies for atomic Hg at 40.81 eV (see Ref.^18^ and refs. therein). For the data sets at 90 and 180 eV from BESSY-II, the double ionisation and autoionisation energies of Hg were used as internal calibrants.

### Computational details

The potential energy curves of the electronic states of S$_2^{2+}$ in the singlet, triplet, quintet and septet spin multiplicities were computed using highly correlated ab initio methods, such as the state-averaged complete active space self-consistent field (SA-CASSCF) approach^30,31^ followed by the Multi-Reference Configuration Interaction (MRCI) method^32–34^ as implemented in MOLPRO 2015^35,36^. For the description of the sulfur atom, we used the aug-cc-pV(5+d)Z basis set^37–39^. The inclusion of the tight-d functions is necessary for a good description of the sulfur atoms. From this, the energetic profiles for the dissociation of the dication can be computed, including both the S$^{+}$ fragments in their ground states ($^{4}$S$_{\mathrm {u}}$) and/or where one of the two asymptotes for the sulfur ions are in an excited state ($^{2}$D$_{\mathrm {u}}$ and/or $^{4}$S$_{\mathrm {g}}$). Also, using the restricted coupled cluster single-double with perturbative treatment of triple excitations (RCCSD(T)) level of theory^40–42^ with the same basis set, the double ionisation energy of S$_2$ and the lowest dissociation limits of S$_2^{2+}$ have been determined. The basis set superposition error (BSSE) correction has been included, but amounted to only 0.01 eV. For the states having a potential well, their potential energy curves was incorporated into nuclear motion treatment to deduce their rotational and vibrational spectroscopic constants using the method of Cooley^43^ and the derivatives at the minimum energy distances and standard perturbation theory. For validation of the present approach, we computed the potential energy curves of S$_2$ and S$_2^+$ in their electronic ground states. The results are given in Table 2, together with a comparison to available experimental data. As can be seen there is a good agreement with the experimental results. Also, we computed an adiabatic single ionisation energy of S$_2$ of about 9.26 eV which is close to the known experimental value of 9.38 eV^10^. Therefore, the computed data for S$_2^{2+}$ dication should be of similar accuracy.

**Table 2:** Spectroscopic constants of the ground electronic states of S$_2$ (X$^{3}\Sigma _{\mathrm {g}}^{-}$) and S$_2^+$ (X$^{2}\Pi _{\mathrm {g}}$). The constants provided are the equilibrium distance R$_e$, the harmonic wavenumber $\omega _e$, the anharmonic terms $\omega _e x_e$ and $\omega _e y_e$, and the rotational constants B$_e$ and $\alpha _e$.

State		R$_e$ (Bohr)	$\omega _e$ (cm$^{-1}$)	$\omega _e x_e$ (cm$^{-1}$)	$\omega _e y_e$ (cm$^{-1}$)	B$_e$ (cm$^{-1}$)	$\alpha _e$ (cm$^{-1}$)
S$_2$ (X$^{3}\Sigma _{\mathrm {g}}^{-}$)	Calc.	3.5881	719.8	2.8	0.00271	0.29224	0.00158
	Exp.^44–47^	3.5701	725.65	2.844		0.2954	0.001570
S$_2^+$ (X$^{2}\Pi _{\mathrm {g}}$)	Calc.	3.4630	799.6	3.4	0.00432	0.31374	0.00171
	Exp.^48^	3.4467	806.099	3.3971		0.316974	

## Supplementary Information

Supplementary Table S1.

## Data Availability

The data sets generated during and/or analysed during the current study are available from the corresponding authors on reasonable request.
