# Supplementary material for: An experimental and theoretical characterization of the electronic structure of doubly ionised disulfur
Source: Sci Rep. 2022 Jul 18;12:12236. doi: 10.1038/s41598-022-16327-8 (PMC9293938; doi:10.1038/s41598-022-16327-8)
Supplement: Supplementary file 1 — Supplementary Table S1. [file 41598_2022_16327_MOESM1_ESM.pdf]

Supplementary Information
Double ionisation of disulfur

Emelie Olsson[1], Tarek Ayari[2], Veronica Ideböhn[1], Måns Wallner[1], Richard J. Squibb[1], Jonas Andersson[1], Andreas Hult Roos[1,3], Stefano Stranges[4,5], John M. Dyke[6], John H.D. Eland[7], Majdi Hochlaf[2,*], and Raimund Feifel[1,*]

[1]University of Gothenburg, Department of Physics, Origovägen 6B, 412 58 Gothenburg, Sweden
[2]Université Gustave Eiffel, COSYS/LISIS, 5 Bd Descartes 77454, Champs sur Marne, France.
[3]ELI Beamlines, Institute of Physics AS CR, v.v.i., Na Slovance 2, 182 21 Prague 8, Czech Republic
[4]IOM-CNR Tasc, SS-14, Km 163.5 Area Science Park, Basovizza, 34149, Trieste, Italy
[5]Dipartimento di Chimica e Tecnologie del Farmaco, Universitá Sapienza, Rome, I-00185, Italy
[6]School of Chemistry, University of Southampton, Highfield, Southampton SO17 1BJ, United Kingdom
[7]Oxford University, Department of Chemistry, Physical and Theoretical Chemistry Laboratory, South Parks Road, Oxford OX1 3QZ, United Kingdom
[*]majdi.hochlaf@univ-eiffel.fr, raimund.feifel@physics.gu.se

Computational supplementary information

Table S1. Computed spectroscopic parameters and vertical double ionisation energies (VDIE) of S_2^{2+} states, numbered in order of increasing VDIE. The parameters are the equilibrium distance R_e, the harmonic wavenumber ω_e, the anharmonic terms $\omega_e x_e$ and $\omega_e y_e$, and the rotational constants B_e and α_e.

	State	R_e (Bohr)	ω_e (cm^{-1})	$\omega_e x_e$ (cm^{-1})	$\omega_e y_e$ (cm^{-1})	B_e (cm^{-1})	α_e (cm^{-1})	VDIE (eV)
0	$X\,^1\Sigma_g^+$	3.372	832.2	1.07	0.67	0.33081	0.00199	26.49
1	$1\,^3\Sigma_u^+$	3.853	-	-	-	0.25578	0.00894	28.82
2	$1\,^3\Delta_u$	3.784	568.0	3.58	0.06	0.26271	0.00205	29.7
3	$1\,^3\Sigma_u^-$	3.752	598.1	3.63	0.08	0.26730	0.00178	30.47
4	$1\,^1\Delta_u$	3.747	612.3	5.41	0.25	0.26795	0.00176	30.48
5	$1\,^3\Pi_g$	3.577	-	-	-	0.54900	0.5856	30.85
6	$1\,^1\Sigma_u^-$							30.94
7	$1\,^1\Pi_g$	3.632	588.4	2.02	0.32	0.28517	0.00260	31.83
8	$1\,^3\Delta_g$							32.61
9	$2\,^1\Sigma_g^+$	4.250	479.4	2.35	0.05	0.20831	0.00110	32.62
10	$1\,^1\Sigma_u^+$	4.098	275.9	-	-	0.22415	0.00118	33.56
11	$1\,^3\Pi_u$							33.8
12	$1\,^1\Gamma_u$							33.86
13	$1\,^1\Pi_u$							34.22
14	$2\,^3\Pi_u$							34.41
15	$1\,^3\Phi_u$							34.58
16	$2\,^3\Pi_g$							34.86
17	$1\,^1\Delta_g$							35.19
18	$1\,^3\Sigma_g^+$							35.24
19	$2\,^3\Sigma_u^+$							35.27
20	$1\,^1\Phi_u$							35.28
21	$2\,^3\Delta_g$							35.49
22	$3\,^3\Pi_u$							35.56
